# Comparison of Radiomics-Based Machine-Learning Classifiers in Diagnosis of Glioblastoma From Primary Central Nervous System Lymphoma

**DOI:** 10.3389/fonc.2020.01151

**Published:** 2020-09-15

**Authors:** Chaoyue Chen, Aiping Zheng, Xuejin Ou, Jian Wang, Xuelei Ma

**Affiliations:** ^1^State Key Laboratory of Biotherapy and Cancer Center, West China Hospital, Sichuan University, Collaborative Innovation Center for Biotherapy, Chengdu, China; ^2^Department of Neurosurgery, West China Hospital, Sichuan University, Chengdu, China; ^3^West China School of Medicine, West China Hospital, Sichuan University, Chengdu, China; ^4^School of Computer Science, Nanjing University of Science and Technology, Nanjing, China; ^5^Department of Biotherapy, Cancer Center, West China Hospital, Sichuan University, Chengdu, China

**Keywords:** glioblastoma, primary central nervous system lymphoma, magnetic resonance imaging, radiomics, machine learning

## Abstract

**Purpose:** The purpose of the current study was to evaluate the ability of magnetic resonance (MR) radiomics-based machine-learning algorithms in differentiating glioblastoma (GBM) from primary central nervous system lymphoma (PCNSL).

**Method:** One-hundred and thirty-eight patients were enrolled in this study. Radiomics features were extracted from contrast-enhanced MR images, and the machine-learning models were established using five selection methods (distance correlation, random forest, least absolute shrinkage and selection operator (LASSO), eXtreme gradient boosting (Xgboost), and Gradient Boosting Decision Tree) and three radiomics-based machine-learning classifiers [linear discriminant analysis (LDA), support vector machine (SVM), and logistic regression (LR)]. Sensitivity, specificity, accuracy, and areas under curves (AUC) of models were calculated, with which the performances of classifiers were evaluated and compared with each other.

**Result:** Brilliant discriminative performance would be observed among all classifiers when combined with the suitable selection method. For LDA-based models, the optimal one was Distance Correlation + LDA with AUC of 0.978. For SVM-based models, Distance Correlation + SVM was the one with highest AUC of 0.959, while for LR-based models, the highest AUC was 0.966 established with LASSO + LR.

**Conclusion:** Radiomics-based machine-learning algorithms potentially have promising performances in differentiating GBM from PCNSL.

## Introduction

Glioblastoma (GBM) and primary central nervous system lymphoma (PCNSL) are considered as the common primary brain tumors, which share similar radiological characteristics but diverse in therapeutic strategies (1–3). The standard of treatment for a GBM is total resection, followed by daily radiation and chemotherapy (like temozolomide) for 6.5 weeks, then a 6-month regimen of oral chemotherapy given 5 days a month, while the first-line treatment for PCNSL is systemic chemotherapy (like high-dose methotrexate regimen) (4). In most cases, the morphological description of two types of tumors on MRI is characteristic enough for adequate discrimination (5, 6). However, misdiagnosis could still incur in some cases because the images of atypical GBM and atypical PCNSL could mimic each other (7). Advanced MRI technology could be useful in the differentiation. However, the urgency of novel radiological methods focused on conventional MR sequences has still been highlighted given that the advanced MRI cannot be performed as the routine examination for every patient.

Texture analysis (TA) refers to a number of a set of mathematical methods describing the features of images, with which non-visual information could be represented with analyzable pixel intensities and the spatial distributions (8, 9). It has been applied as the radiological imaging biomarkers to evaluate tumor heterogeneity, and showed promising ability in as tumor diagnosis, presurgical grading, as well as gene mutation prediction (10–12). Moreover, with quantified analyses of images, it has also been incorporated with various novel computer technologies, such as machine learning (13–16).

The purpose of the present study is to discriminate GBM from PCNSL with radiomics-based machine-learning algorithms in contrast-enhanced T1-weighted (T1C) imaging. In addition, we evaluated different combinations of selection methods and classifiers, trying to make comparison of models' performances.

## Method

### Patient Selection

The patients were selected from neurosurgery department by reviewing the electronic medical records between 2015 and 2018. The including criteria of patients were as follows: (1) pathologically confirmed on GBM or PCNSL; (2) undertook MR scan before any tumor biopsy or surgery; (3) newly diagnosed GBM or PCNSL. Some patients were excluded because of the history of intracranial surgery or irrelevant intracranial diseases. In total, 138 patients (72 men, median age 48 years; and 66 women, median age 54 years) were enrolled from the institution database, including 76 patients diagnosed with GBM and 62 diagnosed with PCNSL.

**Figure 1:**
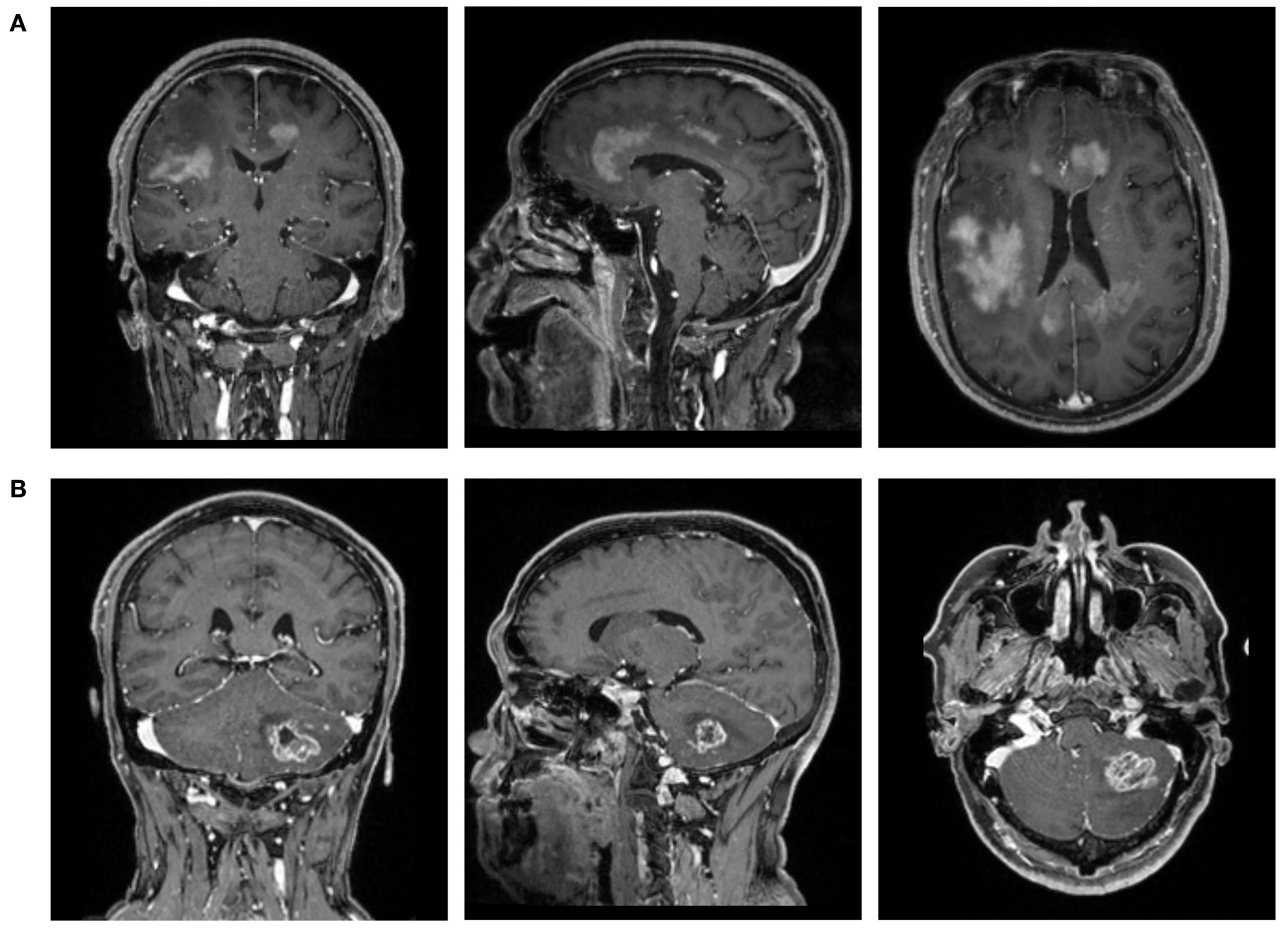
The magnetic resonance images (T1C) of patients with **(A)** primary central nervous system lymphoma (PCNSL) or **(B)** glioblastoma (GBM).

The MR images were collected from the PACS system in the radiological department. We focused on conventional MR sequences, including T1-weighted image (T1WI), contrast-enhanced T1-weighted (T1C) imaging, T2-weighted image (T2WI), and fluid-attenuated inversion recovery, considering that the advanced MR sequences were not commonly used in our institution. After the initial evaluation of images, T1C was selected as the study sequences with rather clear description of the boundary between the tumor tissues and normal brain tissue (Figure 1).

### MRI Protocol

The preoperative MR scan was conducted with 3-T GE MRI system with an eight-channel phase-array head coil. The protocols of the contrast-enhanced T1-weighted imaging were time repetition = 2,000 ms, field of view = 240 × 240 mm^2^, time echo = 30 ms, 30 axial slices, slice thickness = 5 mm (no slice gap), flip angle = 90°, and 200 volumes in each run. Gadopentetate dimeglumine (0.1 mmol/kg) were taken as the contrast agent. The multi-directional data of contrast-enhanced MRI were collected with the continuous interval time of 90–250 s.

All procedures involving human participants were in accordance with the ethical standards of the institutional and/or national research committee. The Ethics Committee of Sichuan University approved this retrospective study. Written informed consent was necessary before radiological examination (written informed consent for patients <16 years old was signed by parents or guardians) for all patients. They agreed to undertake the examination if needed and were informed that the statistics (including MR image) might be used for academic purposes in the future.

### Texture Feature Extraction

Two neurosurgeons participated in the extraction of texture features by using lifeX software (http://www.lifexsoft.org) under the supervisions of senior radiologists. By manually drawing along the tumor tissue slice by slice, the software automatically retrieved 3D-based texture features from two sets of orders with default settings (17). In the first order, statistics from shape- and histogram-based matrix were retrieved. In the second order, statistics from gray-level co-occurrence matrix (GLCM), gray-level zone length matrix (GLZLM), neighborhood gray-level dependence matrix (NGLDM), and gray-level run length matrix (GLRLM) were retrieved. The images were excluded of which the volume of interest did not reach 64 voxels to avoid the interference of the lower image matrix resolution.

Mann–Whitney *U*-test was employed to explore if there is significant statistical difference between the data extracted by two researchers. The results suggested that none of the features were significantly different, implying that the results could be considered reliable and reproducible (shown in Supplementary Material 1).

### Classification Algorithm Application

The patients were randomly divided into the training group and the validation group on the proportion of 4:1. For machine-learning classifiers, the optimal texture features were selected first for classifiers to reduce the number of input variables to improve the performance of the model and to both reduce the computational cost. Considering the optimal selection method was controversial for different classifiers, five methods were conducted separately, including distance correlation, random forest (RF), least absolute shrinkage and selection operator (LASSO), eXtreme gradient boosting (Xgboost), and Gradient Boosting Decision Tree (GBDT).

**Figure 2:**
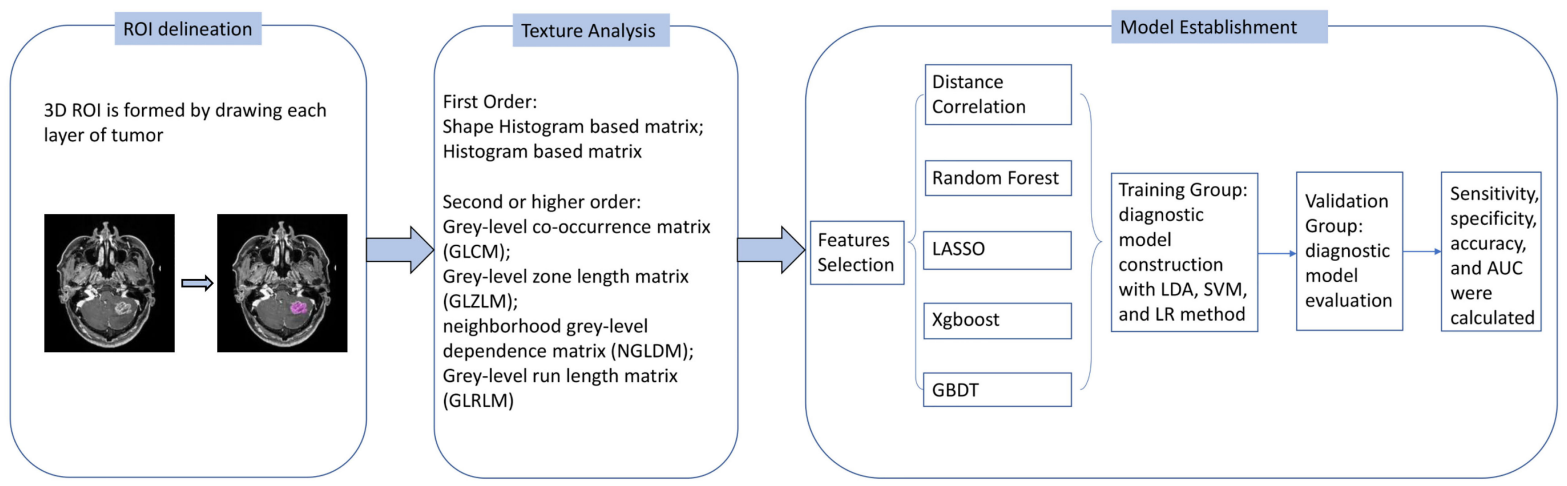
Flow chart of image processing and machine learning.

The purpose of machine learning was to establish and train the models to discriminate GBM from PCNSL with radiomics features extracted from T1C imaging. Three classifiers were tested, including linear discriminant analysis (LDA), support vector machine (SVM), and logistic regression (LR). Thus, 15 diagnostic models were evaluated with different combinations of selection methods and classifiers. The models were trained with the statistics of the training group and tested in the validation group. Sensitivity, specificity, area under the receiver operating characteristic curve (AUC), and accuracy of each model were recorded for evaluation. On application of each model, the cycle of training-validation was performed 100 times to obtain the realistic distribution of classification accuracies. The flow chart of the study is represented in Figure 2.

The models were programmed using Python Programming Language in this study. The models were directly established with default hyperparameter settings of scikit-learn packages (https://scikit-learn.org/stable/).

## Result

The selected features with different methods are represented in Table 1. Four features, GLRLM_LGRE, GLRLM_HGRE, GLRLM_SRHGE, and GLZLM_HGZE, were almost selected even using different methods, suggesting that they were the most significant features in discrimination compared with the others. The other selected features should be reasonably considered as relevant in discrimination, but was hard to tell how much they influenced the algorithms' performances.

**Table 1:** The features selected with different methods.

**Selection method**	**Selected features**
Distance correlation	GLRLM_LGRE; GLRLM_HGRE; GLRLM_SRLGE; GLRLM_SRHGE; GLRLM_LRLGE; GLZLM_LGZE; GLZLM_HGZE; GLZLM_SZLGE
RF	GLRLM_LGRE; GLRLM_HGRE; GLRLM_SRLGE; GLRLM_SRHGE; GLRLM_LRHGE; GLZLM_HGZE
LASSO	minValue; meanValue; stdValue; SHAPE_Volume; GLCM_Contrast; GLRLM_HGRE; GLRLM_SRHGE; GLRLM_LRHGE; GLRLM_GLNU; GLRLM_RLNU; GLZLM_LZE; GLZLM_HGZE; GLZLM_SZHGE; GLZLM_LZHGE; GLZLM_GLNU; GLZLM_ZLNU
XgBoost	GLRLM_LGRE
GBDT	GLRLM_LGRE; GLRLM_HGRE; GLRLM_SRLGE; GLRLM_SRHGE; GLRLM_LRHGE; GLZLM_LGZE; GLZLM_HGZE; GLZLM_SZLGE; GLZLM_SZHGE

*RF, random forest; LASSO, least absolute shrinkage and selection operator; Xgboost, eXtreme gradient boosting; GBDT, Gradient Boosting Decision Tree*.

The performances of models are listed in Table 2. As mentioned previously, the models were established with different combinations of selection methods and classifiers. The results indicated that all three classifiers represented impressive differential ability when using suitable selected features, and the LDA classifier showed much better compatibility compared with other classifiers. Over-fitting was observed in six models, including RF + SVM, Xgboost + SVM, GBDT + SVM, and RF + LR, Xgboost + LR, and GBDT + LR. For LDA-based models, the AUCs in the validation group were 0.978, 0.964, 0.977, 0.750, and 0.956; for the SVM-based models, the AUCs were 0.959 and 0.822; and for LR-based models, the AUCs were 0.933 and 0.975.

**Table 2:** Results of the discriminative model in distinguishing GBM from PCNSL in the training and validation group.

**Classifier**	**Selection method**	**Training group**				**Validation group**			
		**AUC**	**Accuracy**	**Sensitivity**	**Specificity**	**AUC**	**Accuracy**	**Sensitivity**	**Specificity**
LDA	Distance correlation	0.992	0.993	0.996	0.990	0.978	0.979	0.982	0.976
	RF	0.970	0.968	0.935	0.990	0.964	0.957	0.906	0.990
	LASSO	0.997	0.996	0.992	0.995	0.977	0.971	0.955	0.989
	Xgboost	0.791	0.810	0.995	0.740	0.750	0.789	0.995	0.735
	GBDT	0.972	0.970	0.939	0.996	0.956	0.950	0.892	0.995
SVM	Distance correlation	0.957	0.962	0.998	0.934	0.959	0.964	0.997	0.943
	RF (over-fitting)	1	1	1	1	0.5	0.585	1	0.943
	LASSO	0.843	0.835	0.747	0.966	0.822	0.789	0.671	0.965
	Xgboost (over-fitting)	0.5	0.541	0.747	0.967	0.5	0.586	0.671	0.965
	GBDT (over-fitting)	1	1	1	1	0.5	0.586	0.670	0.965
LR	Distance correlation	0.977	0.956	0.961	0.949	0.933	0.927	0.941	0.911
	RF (over-fitting)	1	0.547	1	0.592	0.511	0.515	0.551	0.596
	LASSO	0.959	0.988	0.942	0.981	0.975	0.966	0.975	0.964
	Xgboost (over-fitting)	0.959	0.988	0.942	0.981	0.5	0.5	0.542	0.586
	GBDT (over-fitting)	0.951	0.562	0.954	0.592	0.538	0.515	0.577	0.596

*AUC, area under curve; RF, random forest; LASSO, least absolute shrinkage and selection operator; Xgboost, eXtreme gradient boosting; GBDT, Gradient Boosting Decision Tree; LDA, linear discriminant analysis; SVM, support vector machine; LR, logistic regression*.

**Figure 3:**
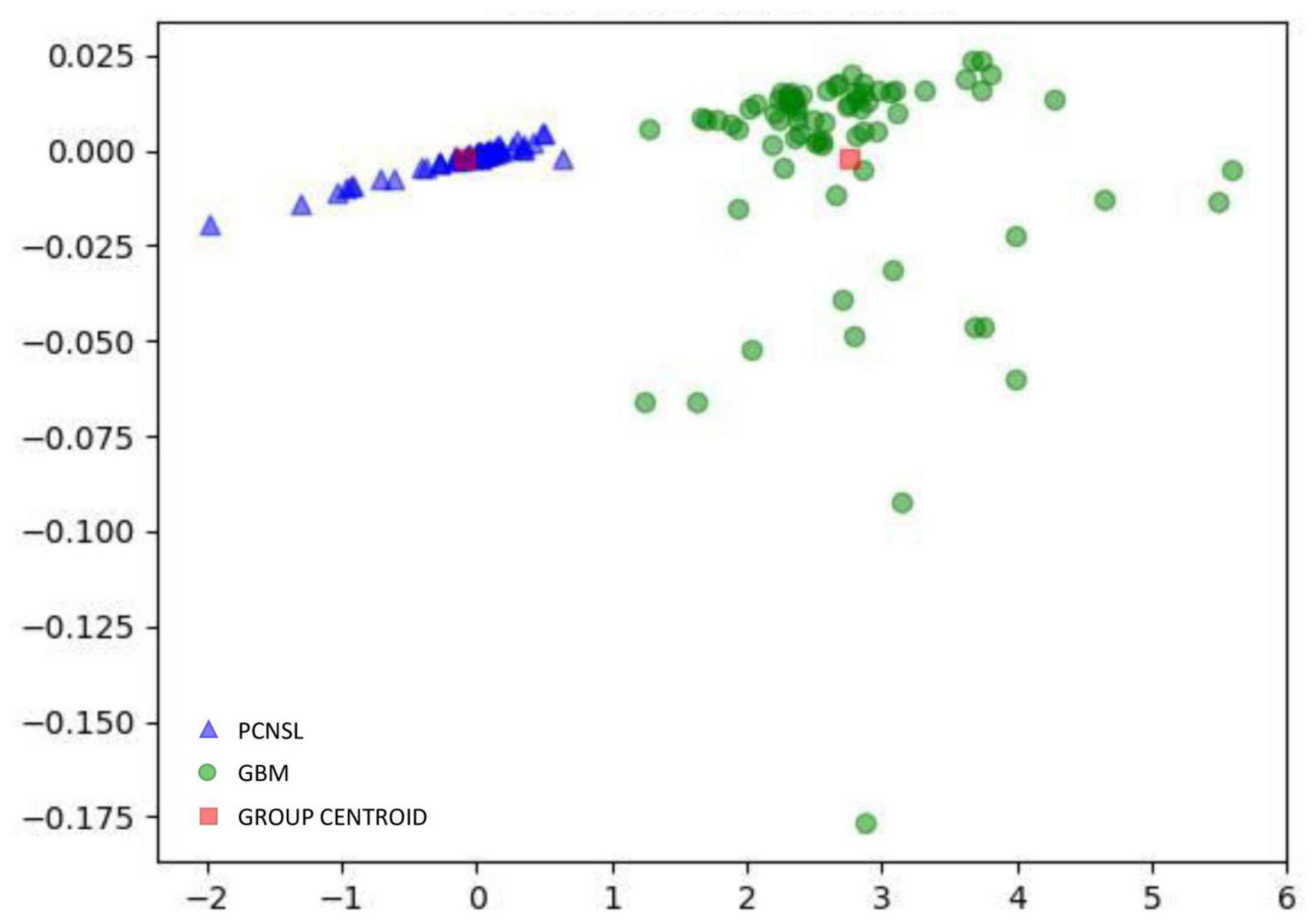
Relationship between the discriminant functions for discriminating GBM from PCNSL.

**Figure 4:**
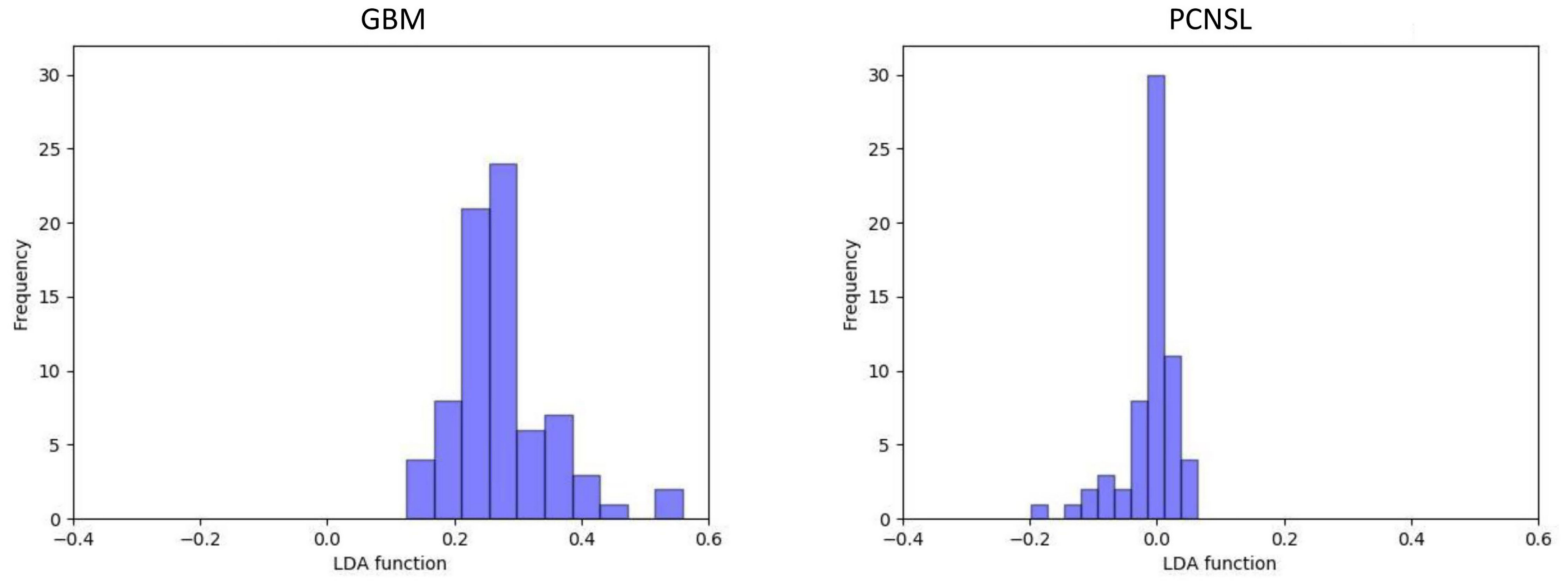
Example of distributions of the LDA function determined for the lesions for one cycle.

In the current study, the optimal model was Distance Correlation + LDA. In the training group, the predictive model showed the discriminative ability with AUC of 0.992, accuracy of 0.993, sensitivity of 0.996, and specificity of 0.990. In the validation group, the performance of the model was rather good, with AUC of 0.978, accuracy of 0.979, sensitivity of 0.982, and specificity of 0.976. The association between discriminative functions from models is represented in Figure 3. Figure 4 represents the examples of distribution of the direct LDA function diagnosis of GBM and PCNSL for one cycle.

## Discussion

In the current study, we performed research in differentiating GBM from PCNSL with the radiomics-based machine-learning technology. Radiomics parameters were extracted from T1C images to detect non-visual information of two types of tumors. The models were established with five selection methods and three classifiers and tested to find the optimal model. The result showed that the radiomics-based machine-learning classifier represented excellent performance in all classifiers with AUC more than 0.900. The optimal model was the combination of Distance Correlation + LDA with AUC of 0.978, accuracy of 0.979, sensitivity of 0.982, and specificity of 0.976. Given that the T1C image was routine examination for GBM and PCNSL, our results suggested that radiomics was a feasible solution for clinical application without requiring additional fees or platform.

Generally, contrast-enhanced T1imaging is a routine radiological examination for patients with GBM or PCSNL. A previous study indicated that at the time of initial presentation for many cases, routine morphological MRI is capable enough in differentiating between GBM and PCNSL lesions. The image patterns are correlated with the tumor characteristics, such as intratumoral hemorrhage, angiogenesis, and necrotic or cystic components. Specifically, heterogeneous enhancement was present in 98.1% of GBM cases and homogenous enhancement in 64.8% of PCNSL cases; necrosis was observed in 88.9% of GBM lesions and 5.6% of PCNSL lesions; multiple lesions were shown in 51.9% of PCNSL cases and 35.2% of GBM cases. Signs of bleeding were uncommon in PCNSL (5.6%) and frequent in GBM (44.4%) (18). Advanced imaging techniques, such as apparent diffusion coefficient (ADC), diffusion-tensor imaging (DTI), dynamic susceptibility-weighted contrast-enhanced MRI, and perfusion weighted imaging, were also additionally performed in discriminating GBM and PCNSL if necessary (19–21). Surgeons could obtain the information on characteristics of tumors to make diagnostic and treatment decisions. However, even with these researches, the differential diagnosis between GBM and PCNSL was still a challenge in some cases, especially given that the conventional MR sequence could only make limited discrimination between two types of tumors and that advanced imaging techniques were not available for all patients.

Comparing with GBM, permeable neovascularization and higher degree of cellularity were more likely to be observed in PCNSL, which theoretically provide the mechanism of TA-based image discrimination (22–24). In our study, radiomics of T1C imaging were used to detect the microscopic differences between GBM and PCNSL, and the results suggested TA was the feasible solution in discriminating GBM and PCNSL radiologically. Radiomics has been reported to distinguish GBM from PCNSL in a previous study, and machine-learning classification model was reported to improve the performance in discrimination (6, 25). Researchers made comparison on diagnostic accuracy between radiologists and machine-learning classifiers, and they suggested that classifiers yielded better diagnostic performance than human radiologists (25). However, the sample sizes of these studies were not large enough and only a few models were tested. Our study enrolled 138 patients with rational proportion of each group and made an evaluation on 15 combinations. In a previous study, RF-based classifier represented perfect performance in discriminating atypical glioblastoma from PCNSL with AUC of 0.98 (6), and SVM-based classifier also represented non-inferior performance to expert human with AUC of 0.877 (25). In our study, the results showed that all three classifiers represented perfect performance when combined with a suitable selection method. It is worth noting that the result of the optimal SVM-based model in our study was with AUC of 0.96, demonstrating much better diagnostic performance than the previous study.

The possible explanation for the improvement was the performance improvement in selection method. Radiomics analysis involved large amounts of features, but machine learning required the most suitable parameters. Previous researchers selected parameters with F-statistic approach into SVM classifier, while we selected with distance correlation, RF, LASSO, Xgboost, or GBDT approach. The combination of LASSO + SVM represented similar discriminative performance such as in the previous study with AUC of 0.822. Besides performances, we can also find that the selection methods were also important to the model stability. Over-fitting is a problem that should be avoided in designing the machine-learning models, which happens when the models catch inaccurate values in the data and the noisy data. Our results suggested that over-fitting probably occurred when using RF, Xgboost, and GBDT as selection methods. Perhaps the features selected with these methods contained too much noise and led to the over-fitting of models.

As for the classifier selection, the purpose of enrollment of three classifiers was to choose the suitable one in discriminating GBM from PCNSL. The results suggested that with suitable features, all of them could represent discriminative ability. It is worthy to note that although we chose Distance Correlation + LDA as the optimal model, some models (like LASSO + LDA and LASSO + LR) also represented pretty similar discriminative performances. The model Distance Correlation + LDA was chosen as the optimal one because it has the minimal difference between sensitivity and specificity compared with LASSO + LDA and LASSO + LR. However, given that all classifier/feature selection methods investigated seem to perform quite comparably and variance in AUC may be partially attributed to small statistical group, the additional gain in information by comparing machine-learning models was quite limited and carefully interpreted. Future investigations with larger sample sizes are required to address this problem and verify our results.

There were several limitations to our study. First, the isolated evaluation of T1C image is not representative of the real clinical work given other sequences (such as ADC, perfusion, DTI, and T2 gradient-echo) could also be useful. Second, the diagnostic performance of radiomics-based machine learning was not compared with other advanced MRI technology. Third, the study cohort is not large enough, requiring study with a large population to verify our results. Forth, the machine-learning classifier was not validated in the other dataset. Considering the considerable variability in images acquired with various MR scanner at different institutions, we cannot guarantee the diagnostic ability of our machine-learning classifier for external datasets. However, the image processing and analysis protocol were open-source packages, meaning they should be validated and reproduced with other datasets.

## Conclusion

Radiomics with machine-learning algorithm technology represented promising ability in differentiating GBM from PCNSL.

## Data Availability Statement

The datasets generated for this study are available on request to the corresponding author.

## Ethics Statement

The studies involving human participants were reviewed and approved by the Ethics Committee of Sichuan University. Written informed consent to participate in this study was provided by the participants' legal guardian/next of kin.

## Author Contributions

XM participated in conceptualization and revised some intellectual content in the manuscript. CC collected MR image, participated in MRI features extraction, and drafted this manuscript. XO collected MR image and participated in MRI features extraction. JW deployed the machine-learning algorism and responsible for statistical analysis. AZ participated in the most revision work. All authors contributed to the article and approved the submitted version.

## Conflict of Interest

The authors declare that the research was conducted in the absence of any commercial or financial relationships that could be construed as a potential conflict of interest.
