# Supplementary material for: Comparison of Radiomics-Based Machine-Learning Classifiers in Diagnosis of Glioblastoma From Primary Central Nervous System Lymphoma
Source: Front Oncol. 2020 Sep 15;10:1151. doi: 10.3389/fonc.2020.01151 (PMC7522159; doi:10.3389/fonc.2020.01151)
Supplement: Supplementary file 1 [file Data_Sheet_1.PDF]

Features	*GBM*	*PCNSL*
minValue	0.40	0.45
meanValue	0.21	0.48
stdValue	0.16	1.00
maxValue	0.26	0.77
HISTO_Skewness	0.31	0.23
HISTO_Kurtosis	0.11	0.48
HISTO_Entropy	0.20	0.19
HISTO_Energy	0.20	0.20
SHAPE_Volume	0.44	0.94
SHAPE_Volumevx	0.42	0.88
SHAPE_Sphericity	0.19	0.30
SHAPE_Compacity	0.09	0.74
GLCM_Homogeneity	0.30	0.18
GLCM_Energy	0.37	0.21
GLCM_Contrast	0.33	0.41
GLCM_Correlation	0.39	0.90
GLCM_Entropy	0.48	0.25
GLCM_Dissimilarity	0.22	0.53
GLRLM_SRE	0.60	0.18
GLRLM_LRE	0.75	0.17
GLRLM_LGRE	0.76	0.46
GLRLM_HGRE	0.66	0.47
GLRLM_SRLGE	0.75	0.46
GLRLM_SRHGE	0.68	0.57
GLRLM_LRLGE	0.62	0.81
GLRLM_LRHGE	0.53	0.32
GLRLM_GLNU	0.37	0.27
GLRLM_RLNU	0.42	0.96
GLRLM_RP	0.64	0.23
NGLDM_Coarseness	0.68	0.78
NGLDM_Contrast	0.23	0.38
NGLDM_Busyness	0.67	0.93
GLZLM_SZE	1.00	0.56
GLZLM_LZE	0.48	0.27
GLZLM_LGZE	0.75	0.49
GLZLM_HGZE	0.82	0.53
GLZLM_SZLGE	0.93	0.23
GLZLM_SZHGE	0.92	0.69
GLZLM_LZLGE	0.53	0.35
GLZLM_LZHGE	0.91	0.30
GLZLM_GLNU	0.28	0.81
GLZLM_ZLNU	0.26	0.56
GLZLM_ZP	0.53	0.20
